# Dermatologic Clues to Emerging Tropical Infections: A Narrative Review for Early Recognition and Differential Diagnosis

**DOI:** 10.7759/cureus.92851

**Published:** 2025-09-21

**Authors:** Gabriela Alejandra Melgar Alvarez, Jenny Tatiana Alarcón Plaza, Irisdey Espinoza Urzua, María Isabel Vidal Vidal, Sebastián Guardiola Segovia, Julio César Flores Rodriguez

**Affiliations:** 1 Internal Medicine, Centro Médico Militar, Guatemala, GTM; 2 General Practice, ESE Hospital San Francisco, Villa de Leyva, COL; 3 Internal Medicine, Universidad Nacional Autónoma de México, Mexico City, MEX; 4 Health Education, Universidad Católica de El Salvador, Santa Ana, SLV; 5 General Practice, Universidad Autónoma de Coahuila, Saltillo, MEX; 6 Aesthetic and Regenerative Medicine, Clínica Aura, San Pedro Garza García, MEX

**Keywords:** chikungunya virus infection, dengue, diagnosis, differential, emerging infectious diseases, exanthema, global health, skin diseases, tropical medicine, zika virus infection

## Abstract

Tropical infections often present with characteristic dermatologic manifestations, which can serve as early indicators for diagnosis. However, these signs are frequently misidentified or underreported in emerging and outbreak settings. This narrative review critically evaluates the diagnostic utility of dermatologic findings across over 25 tropical infections, spanning viral (e.g., dengue, Zika, chikungunya, Mayaro virus, O’nyong-nyong virus), bacterial (e.g., *Bacillus anthracis*, *Mycobacterium ulcerans*, *Rickettsia* spp.), parasitic/helminthic (e.g., *Loa loa*, *Ancylostoma braziliense*, *Tunga penetrans*, *Gnathostoma spinigerum*), and fungal pathogens (e.g., *Fonsecaea pedrosoi*, *Paracoccidioides brasiliensis*). A thematic synthesis was conducted using peer-reviewed case reports, outbreak investigations, and cross-specialty literature bridging dermatology and infectious diseases. Emphasis was placed on lesion patterns, geographic distribution, climate and seasonal influences, and clinical interpretability. Findings indicate that although many infections share overlapping cutaneous features, certain lesion patterns can aid preliminary diagnosis when interpreted in the context of local epidemiology. Limitations include reliance on anecdotal data, nonstandardized terminology, and underinvolvement of dermatologists in outbreak settings. The review concludes that dermatologic signs remain an underutilized but essential tool for early detection. Recommendations include adopting standardized dermatologic terminology, improving clinician training in skin-based recognition, and enhancing collaboration between dermatology and infectious disease specialists. Recognizing cutaneous manifestations can facilitate rapid case identification, optimize public health responses, and improve outbreak preparedness in tropical regions.

## Introduction and background

Tropical infections have been a major challenge to world health, with particular challenges in low- and middle-income countries (LMICs), where amenities have not been well-developed [1]. A characteristic of most of these infections is the manifestation through visible dermatologic manifestations. Such skin symptoms are among the first to appear during the disease, and this is a great chance to detect and treat the disease at an early stage [2]. Although they have diagnostic potential, skin-related signs are commonly ignored in clinical conditions, especially in resource-poor environments where it may not be possible to conduct such laboratory tests in time or the facilities are not present [3].

Dermatologic manifestations are even more important in a diagnostic role in the case of the developing tropical disease. Climate change, the movements of people, urbanization, and deforestation are rapidly shifting the world scene of infectious diseases [4,5]. These factors are spreading the geographic range of vector-borne and zoonotic diseases, even bringing the constrained tropical diseases into other geographic areas. This necessitates the need for clinicians all over the world, including those in non-endemic places, to identify new appearances on the skin that are unfamiliar with new pathogens [6].

In a lot of tropical and subtropical areas, the available and immediate indications of infection are dermatologic [7]. Maculopapular rashes, eschars, petechiae, and other skin lesions may hold the clues that narrow the possibilities in the differential diagnoses, direct the use of empiric therapeutics, and precondition timely action on the part of the authorities to take precautionary steps [8]. Nevertheless, these signs are usually narrow in clinical value due to their lack of specificity and their morphology, which is similar in many pathogens, posing a challenge to the discrete identification of one infection as opposed to another by clinicians on the basis of skin presentation alone [9,10].

Making matters worse in this effort to diagnose is the fact that general medical education has focused little to no attention on dermatologic training, particularly in the primary care and tropical care areas [11]. Consequently, in cases where skin signs are manifested, they may not be interpreted uniformly, resulting in a wrong diagnosis or late treatment [12,13].

Despite numerous case reports and outbreak investigations, there is currently no comprehensive synthesis evaluating the diagnostic utility of dermatologic signs across emerging, re-emerging, and endemic tropical infections. This review fills that gap by critically examining skin manifestations for their specificity, limitations, and contextual relevance in aiding early diagnosis. It draws on peer-reviewed literature, outbreak data, and cross-specialty insights from dermatology and infectious disease fields. To enhance navigability, the review is structured thematically, covering viral, bacterial, parasitic, and fungal infections, with emphasis on characteristic dermatologic patterns, regional and climatic influences, and practical implications for clinical recognition and public health preparedness.

## Review

Methodological approach

This is a narrative literature review that aimed to critically evaluate dermatologic clues to emerging tropical infections. Articles were identified through systematic searches of PubMed, Scopus, Web of Science, and Google Scholar using combinations of keywords such as “tropical infections,” “dermatologic manifestations,” “cutaneous signs,” “emerging infections,” “vector-borne diseases,” and pathogen-specific terms (e.g., dengue, Zika, chikungunya) was conducted for articles published between 2005 and 2025 using relevant keywords. Additional sources included the World Health Organization (WHO) and Centers for Disease Control and Prevention (CDC) reports, outbreak investigations, and cross-disciplinary publications in dermatology and infectious diseases. Reference lists of included articles were also screened to identify relevant studies. Studies were selected based on relevance, clinical focus, and quality. Findings were synthesized thematically, focusing on clinical applicability in tropical medicine. Articles were included if they addressed dermatologic features of emerging or re-emerging tropical infections in human subjects. Non-clinical studies, opinion pieces, and unrelated dermatologic disorders were excluded. The findings were synthesized thematically under categories of viral, bacterial, parasitic, and fungal infections.

Infections were selected based on predefined criteria: (1) emerging, re-emerging, or endemic pathogens in tropical/subtropical regions; (2) documented dermatologic manifestations of diagnostic or epidemiologic significance; and (3) cutaneous signs appearing early in disease that may serve as initial diagnostic cues. Priority was given to vector-borne, zoonotic, and climate-sensitive infections due to their dynamic epidemiology. Limitations include reliance on anecdotal reports, non-standardized terminology, regional differences in reporting, and a paucity of large-scale comparative studies.

Despite these challenges, this review critically appraises dermatologic signs across a broad spectrum of tropical infections, highlighting characteristic patterns, diagnostic considerations, and practical implications. Table 1 summarizes study characteristics, including author/year, tropical region, pathogen/infection type, timing, and utility of skin signs, lesion description, pathophysiology, differential diagnosis, diagnostic tools, practice challenges, and key findings.

**Table 1:** Summary of findings included in review AFB: acid-fast* Bacilli*; AFLP: amplified fragment length polymorphism; ATLL: adult T-cell leukemia/lymphoma; *B. anthracis*:* Bacillus anthracis*; *B. pseudomallei*: *Burkholderia pseudomallei;* CHIKV: chikungunya virus; CLM: cutaneous larva migrans; CMFL: community microfilariae load; CNS: central nervous system; CRP: C-reactive protein; DENV: dengue virus; DHF: dengue hemorrhagic fever; DSS: dengue shock syndrome; EIA: enzyme immunoassay; ELISA: enzyme-linked immunosorbent assay; IFA: immunofluorescence assay; IgG: immunoglobulin G; IgM: immunoglobulin M; LAMP: loop-mediated isothermal amplification; MAT: microscopic agglutination test; MFI: *Mycobacterium ulcerans* infection; NAT: nucleic acid test; nPCR: nested polymerase chain reaction; PCR: polymerase chain reaction; PRNT: plaque reduction neutralization test; RNA: ribonucleic acid; RT-PCR: reverse transcription polymerase chain reaction; SFG: spotted fever group; TB: tuberculosis; VD: visceral disease; WHO: World Health Organization

Author/year	Tropical region	Pathogen & infection type	Timing & utility of skin signs	Rash/lesion description	Pathophysiology/mechanism	Differential diagnosis & misdiagnosis risk	Diagnostic Tools Used	Practice Challenges	Key Notes & Quotes
Thomas et al., 2010 [14]	Endemic regions: India, Thailand, Latin America. Vector:* Aedes aegypti *(tropical/subtropical). Seasonality: peaks in rainy seasons. Co-circulating pathogens: chikungunya, leptospirosis	Dengue virus (viral)	Early (24-48 h): flushing erythema; late (3-6 days): maculopapular rash. Skin signs are moderately specific but critical for early diagnosis	Morbilliform/maculopapular, confluent erythema, petechiae, "white islands in a sea of red"; starts on face/dorsum, spreads centrifugally	Viral interaction with Langerhans cells. Immune-mediated capillary changes. No direct viral invasion in the skin	Broad DDx: Chikungunya, measles, rubella, and scarlet fever. High misdiagnosis risk due to overlapping symptoms in the tropics.	Histopathology (perivascular edema) Immunofluorescence (often negative) Tourniquet test (>20 petechiae/2.5 cm²).	Overlap with drug rashes Lab confirmation is often delayed in resource-limited areas. Travel clinic challenges.	Recognition of DF rash permits rapid early diagnosis. critical as DF can progress to DHF or DSS."
Bhat et al., 2011 [15]	Location: Coastal Karnataka, India (Mangalore). Climate: humid tropical (monsoon-driven). Epidemiology: 2008 epidemic (39,042 suspected cases). Risk factors: high *Aedes *breeding (water stagnation), urban/rural transmission. Co-circulating pathogens: dengue, Zika (diagnostic overlap). Unique environmental links: UV-induced pigmentation, heat exacerbating desquamation	Chikungunya virus (CHIKV, Alphavirus)-mosquito-borne (*Aedes aegypti/albopictus*)	Early (1-7 days): maculopapular rash (52%), aphthous ulcers (22.66%). Late (1-2 weeks): pigmentary changes (16%), desquamation (10.66%). Utility: critical for early diagnosis in resource-limited tropical settings	1. Morbilliform/maculopapular rash (trunk/limbs/face). 2. Aphthous ulcers (oral/groin/axillae-male-specific). 3. Hyper/hypopigmentation (sun-exposed areas). 4. Desquamation (face/palms/soles). 5. Exacerbations (psoriasis/eczema). 6. Rare: scrotal dermatitis, necrotic ulcers	Rash: viral replication in skin + immune-mediated vasculitis. Ulcers: direct viral cytotoxicity + cytokine storm. Pigmentation: post-inflammatory melanocyte activation + tropical UV exposure. Desquamation: keratinocyte damage from prolonged fever	High misdiagnosis with dengue (37%), measles (12%), and drug rash (8%). Distinctive Features: Groin ulcers (CHIKV-specific in males), centrofacial pigmentation	Primary: Clinical (WHO "suspect case" criteria) Lab: IgM ELISA (limited availability), dengue serology exclusion Histopathology: Non-specific perivascular lymphocytosis	1. Overlap with dengue in tropical fever clusters 2. Male genital ulcers mistaken for STIs 3. Delayed lab confirmation (avg. 7-10 days in tropics) 4. Photosensitivity complaints requiring dermatology referral	Intertriginous ulcers are pathognomonic for CHIKV in tropical males. Facial desquamation—a novel tropical variant linked to sun exposure. Pigmentation patterns mirror malaria/dengue in this region
Paniz-Mondolfi et al., 2018 [16]	Primary regions: Americas (Venezuela, Brazil, Caribbean), Southeast Asia, Pacific Islands. Regional features: Venezuela: proximal arm/leg rash accentuation. Brazil: high rates of congenital syndrome. French Polynesia: first major outbreak (2013-2014). Caribbean: co-circulation with DENV/CHIKV	Zika virus (Flavivirus, mosquito-borne)	Onset: 24-48 h after symptoms (90% of cases). Diagnostic utility: early maculopapular rash is pathognomonic. Conjunctivitis + rash = high suspicion. Duration: 3-10 days of incubation, ~1 week of illness	Classic presentation: pruritic maculopapular rash (face → trunk → palms/soles), blanching erythema, reticular/net-like pattern. Atypical manifestations: petechiae/ecchymosis (thrombocytopenia), psoriatic-like plaques post-infection, periarticular edema (wrists/ankles), mucosal ulcers/jaundice. Regional variation: Venezuelan cases show proximal limb accentuation	Transmission: *Aedes *mosquito bite → skin inoculation. Cellular targets: keratinocytes (AXL receptor-mediated), dermal fibroblasts, Langerhans/dendritic cells ("Trojan horses"). Immune evasion: apoptotic blebs shield the virus. Autophagy induction in fibroblasts. Mosquito factors: saliva proteins enhance replication	High-Confidence Mimics: Dengue (later rash onset) Chikungunya (similar arthralgia) Mayaro virus Other Considerations Rubella/measles HHV-6/parvovirus B19 Enteroviruses Misdiagnosis Risks: 60-70% asymptomatic cases Co-infections with DENV/CHIKV and drug rash overlap	First-Line: NAT (blood/serum/urine) ≤7 days Histopathology: Perivascular lymphocytic infiltrate Spongiosis/dyskeratosis Psoriasiform hyperplasia (post-ZIKV) Serology Caveats: IgM requires PRNT confirmation. Cross-reactivity with other flaviviruses	Clinical: Distinguishing DENV/CHIKV/ZIKV triads Recognizing psoriatic flares Thrombocytopenia monitoring Laboratory: Short NAT detection window IgM persistence (months) Public Health: Pregnancy complications Co-epidemic surveillance	The skin provides pathognomonic clues despite significant overlap with other arboviruses. Venezuelan cases uniquely demonstrate proximal extremity rash accentuation. NAT remains the gold standard but must be performed early (<7 days).
Kim et al., 2025 [17]	Endemic: Americas (Mexico, Brazil), Middle East (Syria, Lebanon), Central Asia, North Africa. Emerging: Southern Europe, refugee camps in temperate zones. Climate impact: expanded sandfly range due to warming (20-30°C optimal), increased rainfall. Key species: *L. braziliensis* (mucocutaneous), *L. major* (desert sores),* L. mexicana* (self-healing)	Cutaneous leishmaniasis (*Leishmania *spp., sandfly-borne)	Early: papules/nodules (2-8 weeks post-bite). Late: ulcers with raised borders (diagnostic but often missed). Utility: critical for early diagnosis in resource-limited tropical settings	Localized: painless ulcers with "volcano" edges (face/limbs). Diffuse: non-ulcerative nodules. (immunocompromised). Tropical variants: Larger necrotic ulcers (*L. braziliensis*), dry plaques (*L. major*)	Sandfly bite → macrophage infection → Th1/Th2 imbalance Tropical adaptation: Enhanced parasite survival in humid climates Strain-specific immune evasion (e.g., L. braziliensis inhibits macrophage ROS)	High-risk misdiagnoses: Tropical: Fungal infections, mycobacterial ulcers Non-tropical: Skin cancer, sarcoidosis Contributors: Clinical overlap in tropics, lack of diagnostic tools	Microscopy (Giemsa-stained smears) - 80% sensitive in fresh ulcers PCR (gold standard but scarce in tropics) AI/telemedicine (emerging in tropical LMICs)	Tropical barriers: Heat-labile medications, no cold chain Refugee camps: Overcrowding → sandfly proliferation Treatment access: Miltefosine often unavailable	CL cases in Lebanon surged 230x post-Syrian refugee crisis due to tropical strains in camps. Imported L. mexicana cases in Germany linked to Yucatán travel WHO: Only Texas reports CL cases in U.S. despite tropical spread"
Dori et al., 2012 [18]	Location: Anfilo District, West Wellega, Ethiopia (750 km west of Addis Ababa). Climate: tropical riverine zone with perennial rivers (Eda, Ega). Vector: *Simulium damnosum* complex, breeding in fast-flowing waters. Endemicity: hyperendemic (74.8% MF prevalence)	*Onchocerca volvulus* (filarial nematode). Transmission: tropical blackfly vector. Tropical Impact: 90% of global cases occur in tropical Africa; Ethiopia has 3M infected	Chronic tropical exposure: skin signs (leopard skin, nodules) develop over the years in endemic zones. Diagnostic utility: critical in tropical field settings where labs are scarce	Tropical dermatopathology: leopard skin (19.1%): hypopigmented macules from chronic tropical exposure. Nodules (12.1%): subcutaneous, palpable in tropical populations. Pruritus (64.3%): hallmark of tropical filariasis	Tropical ecology: microfilariae migrate to the skin via blackfly bites (tropical vector). Immune response to MF causes depigmentation (leopard skin), unique to tropical strains. Nodules form around adult worms in subcutaneous tissue	Tropical Overlap: Leprosy (similar hypopigmented lesions). Lymphatic filariasis (co-endemic in tropics). Scabies (pruritus mimic). Risk: High in tropical clinics due to overlapping endemic diseases.	Tropical Adaptations: Skin snip microscopy (field-friendly in tropics). CMFL (logarithmic scale for high-burden tropical areas). Clinical nodule palpation (limited utility in Ethiopia vs. West Africa).	Tropical Barriers: Nodule-based WHO protocols underestimate MF prevalence in Ethiopia. Self-treatment with ivermectin is common in tropical communities. Farmers (82.1% infected) were exposed near blackfly habitats.	Tropical Significance: 74.8% MF prevalence signals hyperendemicity in tropical riverine zones. Leopard skin (19.1%) correlates with age (R²=0.79), reflecting chronic tropical exposure. CMFL of 19.6 demands sustained CDTI in tropical hotspots.
Denk et al. 2016 [19]	Endemic regions: Eastern Anatolia (Turkey), Iran, Pakistan, and Africa. Risk factors: animal husbandry, slaughtering. Seasonality: peaks in arid seasons (July-Oct). Co-circulating pathogens: *Staphylococcus*/*Streptococcus *(secondary infections)	*Bacillus anthracis* (bacterial)	Incubation: 1-7 days post-exposure. Early sign: painless papule → Late (24–48 h): vesicle → day 3+: black eschar. Skin signs are critical for early diagnosis in endemic areas	Malignant pustule (59%): papule → ulcer with black eschar. Malignant edema (41%): severe swelling, necrosis (face/neck). Secondary bacterial infection (33.3%)	Toxin-mediated (edema/lethal factors). Spores enter via skin breaks. No direct bacterial invasion in late lesions	Broad DDx: Plague, tularemia, staphylococcal lymphadenitis, orf, or Buruli ulcer. High misdiagnosis risk in non-endemic areas due to rarity.	Gram stain (64% detection). Culture (38.5% yield). Clinical history (animal contact).	Antibiotic pretreatment obscures lab results. Lack of clinician experience in urban settings. Bioterrorism concerns complicate diagnosis.	Cutaneous anthrax lesions vary widely; lack of experience in non-agricultural settings delays diagnosis. Mortality: 2.5% (laryngeal edema/sepsis).
Thipmontree, Wilawan, et al. 2016 [20]	Location: South Korea (Gwangju, Haenam, Jangheung). Climate: temperate with monsoonal influence (autumn peak incidence). Epidemiology: 2005 outbreak (176 confirmed cases). Risk factors: farming activities, mite exposure in grasslands. Unique findings: gender-specific eschar distribution linked to clothing pressure (brassieres in females)	*Orientia tsutsugamushi* (Gram-negative bacterium)-mite-borne (*Leptotrombidium larvae*)	Early (3-10 days post-bite): eschar formation (92% of cases) with surrounding erythema. Utility: pathognomonic for diagnosis; PCR/IFA confirmation from eschar has 100% sensitivity	Eschar: Black necrotic center with erythematous halo (5–20 mm). Gender-specific distribution: males: below the umbilicus (35.8%), lower limbs (22.6%). Females: front chest above the umbilicus (40.7%), back (15.7%). Atypical variants: axillary/perineal ulcers (no necrosis in damp areas)	Eschar: mite bite inoculates* O. tsutsugamushi* → local proliferation → vasculitis + necrosis. Gender disparity: clothing pressure (brassieres/underwear) creates warm/damp microenvironments, favoring mite attachment	High misdiagnosis with: Rickettsialpox, anthrax (eschar-like lesions). Key distinguishers: Lymphadenopathy near eschar (79.5% of cases). Geographic exposure history (endemic tropics).	Gold standard: IFA (IgM ≥1:80 or 4-fold titer rise). Eschar PCR: 100% sensitivity for early diagnosis. Exclusion: Dengue/measles serology.	1. Eschar painless/small → overlooked (especially in dark-skinned patients). 2. Atypical ulcers mimic STIs (females) or trauma. 3. Strain variability affects eschar prevalence (Boryong vs. Karp strains).	Front chest eschar in females and infra-umbilical eschar in males are diagnostic hallmarks in endemic zones. PCR on eschar crust enables diagnosis even after antibiotic treatment.
Sakyi et al., 2016 [21]	Endemic regions: West Africa (Ghana, Benin, Togo), Central Africa (Cameroon, DRC), Australia, Southeast Asia. Regional features: Ghana: high burden; co-circulation with TB. Australia: low case numbers but severe lesions. Benin/Togo: rural hotspots near water bodies	*Mycobacterium ulcerans* (bacterial, necrotizing)	Early stage (pre-ulcerative): firm, nontender nodule/plaque (weeks to months). Ulcerative stage: undermined edges, necrotic base (pathognomonic). Utility: skin signs are critical for early diagnosis to prevent disability	Pre-ulcerative: nodules/plaques with induration, non-painful edema. Ulcerative: necrotic ulcers with "undermined edges," satellite lesions osteomyelitis in advanced cases. Healing stage: contractures/scarring	Toxin-mediated: mycolactone causes necrosis and immunosuppression. Transmission: environmental exposure (water/insect bites), skin inoculation → local proliferation → systemic spread. Histopathology: necrotic subcutaneous fat, clustered acid-fast bacilli (AFB)	High-Confidence Mimics: Cutaneous TB Leprosy (borderline cases) Chronic fungal infections Misdiagnosis Risks: 30% false positives in microscopy Overlap with other mycobacterial infections	Gold Standard: - IS2404 PCR (92–100% sensitivity) Routine: Microscopy (ZN staining; 40–78% sensitivity) Histopathology (90% sensitivity) Emerging: LAMP assays (field-friendly) Mycolactone detection (TLC/FTLC)	Clinical: Delayed diagnosis → severe morbidity Distinguishing TB/Buruli co-infections Laboratory: Culture slow (6–12 weeks) PCR contamination risks Access: Limited POC tools in rural areas	Early diagnosis prevents disability; ulcers with undermined edges are pathognomonic. IS2404 PCR remains the gold standard but requires advanced labs. Mycolactone detection could revolutionize POC testing.
Weerakoon et al., 2014 [22]	Sri Lanka (Central Province) endemicity: emerging/re-emerging focus. Climate correlation: humid tropical climate favoring tick vectors. Unique features: no classical eschars (unlike Mediterranean/African variants). High incidence of "fern leaf" necrosis (6% of cases). Ankle arthritis (12%) and facial edema in the elderly	Pathogen: *Rickettsia conorii *(spotted fever group). Infection type: tick-borne rickettsiosis	Onset: rash appears ~3 days post-fever (89% of cases). Diagnostic utility: Maculopapular rash (98.5%) is the primary diagnostic. Clue: fern leaf necrosis pathognomonic but rare (elderly)	Type: 98% maculopapular, 1% macular/papular. Color: erythematous (fair skin) → dusky red (dark skin). Size: 5 mm avg (2-10mm range). Distribution: limbs (81% arms, 67% legs), palms/soles (55-56%). Unique forms: fern leaf necrosis (6%), scrotal gangrene (case report)	Vasculitis: biopsy-confirmed endothelial damage (fibrin thrombi, perivascular lymphocytic infiltrates). PCR confirmation: 17-kDa SFG antigen detected in lesions	High-Confusion Conditions: Dengue/leptospirosis (fever and rash in tropics) Vasculitic disorders (ANCA-associated) Key Differentiators: Tick exposure history (6% had bite marks) Rapid doxycycline response	Serology: IFA (IgM ≥1/32 + IgG >1/256 cutoff) Histopathology: Vasculitis with fibrinoid necrosis Molecular: nPCR for 17-kDa antigen	Tropical Limitations: No eschars (unlike classic SFG) → delayed diagnosis Dusky rash is hard to detect on dark skin. Atypical Cases: Arthritis/edema mimics autoimmune disease. Necrosis/gangrene requires tissue PCR	Fern leaf necrosis is a tropical Sri Lankan variant not described elsewhere. Ankle arthritis and limb rash = SFG in endemic zones "IFA titers >1/256 + vasculitic rash confirm SFG without eschar." *
Singh and Mahmood, 2017 [23]	Endemic areas: Southeast Asia, northern Australia, the Indian subcontinent (Kerala, Karnataka, Vellore), southern China, Hong Kong, and Taiwan. Climate link: heavy monsoons (75-85% of cases in rainy seasons). High-risk groups: farmers, military personnel, and construction workers exposed to tropical soil/water	*Burkholderia pseudomallei* Transmission: percutaneous inoculation/inhalation of contaminated soil/water. Tropical impact: 20% of community-acquired septicemias in northern Australia/Thailand	Acute/tropical presentation: symptoms appear 1-21 days post-exposure (shorter with high inoculum). Chronic/tropical latency: Vietnamese time bomb reactivation after 62 years. Skin abscesses are common but nonspecific in the tropics	Tropical manifestations: cutaneous abscesses (mimicking TB/staph). Suppurative parotitis (children). Visceral abscesses (liver/spleen). Pneumonia (50% of cases, cavitary lesions on X-ray)	Tropical ecology: bacteria thrive in moist tropical soil/water. Inhalation during monsoons or inoculation via wounds. Intracellular survival evades immune response. Risk factors: diabetes/alcoholism (common in tropics)	Tropical Mimics: Tuberculosis (chronic fever/cavitary pneumonia). Staphylococcal abscesses. Leptospirosis (sepsis). High Misdiagnosis Risk: Underreported in India due to overlapping tropical diseases.	Tropical Adaptations: Culture on Ashdown’s medium (selective for tropics). Gram stain ("safety pin" bipolar rods). Serology/PCR (limited in endemic areas due to background antibodies).	Tropical Barriers: Empiric antibiotics often miss coverage (e.g., beta-lactams are ineffective). Lab misidentification as contaminant (Fig. 3–5). High mortality (90% if untreated; 19–35% with therapy).	Tropical Significance: Melioidosis is the ‘great mimicker’ in the tropics, resembling TB/staph. Heavy monsoons drive seasonal outbreaks. Relapse occurs in 13–23% of tropical cases, often fatal.
Leung et al., 2017 [24]	Endemic regions: Central/South America, the Caribbean, Africa, Southeast Asia, and the SE USA. Risk factors: barefoot exposure to contaminated sand/soil (beaches, farms). Seasonality: peak in rainy seasons (15× higher risk). Co-circulating pathogens: Strongyloides, myiasis	*Ancylostoma braziliense *(hookworm larvae, parasitic)	Incubation: 5-15 days. Early sign: pruritic papule → Late (days-weeks): Serpiginous track (2 mm-2 cm/day). Skin signs are pathognomonic but often misdiagnosed in non-endemic areas	Classic CLM: erythematous, serpiginous, raised track (1-4 mm wide). Follicular variant: Pruritic papules/pustules (no central hair). Bullous variant: rare, mimics herpes zoster	Larvae penetrate skin via proteases/hyaluronidase. Confined to the epidermis (lacks collagenase). Hypersensitivity to larval antigens	Broad DDx: Larva currens (Strongyloides), migratory myiasis, scabies, cercarial dermatitis. High misdiagnosis risk (initial diagnosis correct <50%).	Clinical diagnosis (history + track morphology). Dermoscopy (translucent larva/burrows). Confocal microscopy (refractile larvae).	Larval mobility complicates topical treatment. Oral antihelminthics are contraindicated in pregnancy/children. Limited lab confirmation in resource-poor settings.	The pruritic serpiginous track is pathognomonic. Oral ivermectin is the treatment of choice. Complications: Secondary infection, Löffler syndrome (pulmonary eosinophilia).
Heukelbach et al., 2007 [25]	Endemic regions: rural Alagoas State (NE Brazil), Latin America, the Caribbean, and sub-Saharan Africa. Risk factors: barefoot walking, poor sanitation, and sandy soils. Seasonality: higher prevalence in the dry season (29.5% vs. 21.6% in the rainy season)	*Tunga penetrans* (sand flea, ectoparasitic)	Incubation: flea penetration → lesion maturation (days). Early sign: itchy papule. Late (days-weeks): white patch with black dot (egg-expelling flea). Skin signs are critical for diagnosis in endemic areas	Stage I: penetrating flea. Stage II: reddish-brown spot (1–3 mm). Stage III: White patch with black dot (4-10 mm). Stage IV: black crust/necrosis. Common sites: toes (periungual, 70%), soles, and hands (3-6%)	A female flea burrows into the epidermis, hypertrophies, and expels eggs. Secondary bacterial infections (15.5% of cases). Inflammation due to flea antigens and bacterial superinfection	Broad DDx: Scabies, cutaneous larva migrans, myiasis, bacterial abscess. High misdiagnosis risk in non-endemic areas due to rarity.	Clinical diagnosis (visual inspection of pathognomonic lesions). Fortaleza classification (stages I–IV). Rarely: Biopsy (if atypical presentation).	High reinfestation rates in endemic areas. Limited access to treatment in resource-poor settings. Severe morbidity (nail loss, difficulty walking) in chronic cases.	Tungiasis is a neglected disease of poverty, with children bearing the highest burden. Severe cases: Deep fissures (10.5%), nail loss (5.5%), and tetanus risk. Prevention: Footwear, improved sanitation, and flea control.
Nagraik et al., 2020 [26]	Endemic in India (Mumbai, Kerala, and Andaman), Thailand, Latin America, and the Caribbean. Outbreaks follow floods/monsoons (e.g., Nicaragua 1996, Mumbai 2005)	Bacterial spirochete:* Leptospira interrogans*. Reservoirs: rodents, livestock (cattle, pigs). Transmission: contaminated water/soil via skin/mucosal contact	Conjunctival suffusion (early, 3-5 days) is pathognomonic but often missed. Petechiae/jaundice (late, severe Weil's disease). Skin signs are less diagnostic than systemic symptoms (fever, renal failure)	Non-specific: rare maculopapular rash. Icteric form: yellowing (jaundice) + petechiae. Hemorrhagic: pulmonary bleeding (no classic rash)	Toxins: LPS (atypical TLR2 activation), hemolysins (RBC lysis). Immune evasion: resists complement, binds ECM proteins (e.g., LipL32). Organ damage: capillary leakage → renal/hepatic failure	Common misdiagnoses: dengue, malaria, hepatitis, and scrub typhus. Overlap: Fever, jaundice, and thrombocytopenia. Key distinguisher:Conjunctival suffusion (absent in dengue/malaria).	Gold standard: Microscopic Agglutination Test (MAT)—but slow, requires live cultures. Rapid tests: IgM ELISA (early), PCR (DNA detection). Ancillary: Dark-field microscopy (low sensitivity).	Resource limitations: MAT is impractical in rural areas. Cross-reactivity: Serology confounded by prior exposure/vaccination. Culture difficulty: Fastidious growth (weeks).	Leptospirosis is the most underdiagnosed zoonosis due to non-specific presentation and lack of affordable diagnostics. High-risk groups: farmers, flood-affected communities, and slum dwellers.
Alladio et al., 2024 [27]	Endemic regions: West/Central Africa (*Loa loa*), sub-Saharan Africa/Central & South America (*Mansonella perstans*). Vector: *Chrysops *flies (*Loa loa*), *Culicoides *midges (*Mansonella*)	Filarial nematodes: *Loa loa *(loiasis), *Mansonella perstans* (mansonellosis)	Loiasis: transient calabar swelling (allergic angioedema, hours to 7 days), eyeworm migration (conjunctival). Mansonellosis: chronic pruritus, urticaria, transient edema (nonspecific timing)	Loiasis: Calabar swelling: localized, allergic angioedema. Eyeworm: subconjunctival worm migration. Mansonellosis: pruritus, urticaria, rash, and lymphadenopathy	Loiasis: immune response to migrating adult worms/microfilariae. Mansonellosis: Immune-mediated reactions to microfilariae/adults in body cavities	Loiasis: Misdiagnosed as other filariasis (e.g., onchocerciasis), splenic nodules confused with malignancies. Mansonellosis: Overlaps with other helminth infections (e.g., strongyloidiasis).	Microscopy: Blood smears (daytime for Loa loa), Knott’s/Nucleopore filtration. Serology: Pan-filarial ELISA (cross-reactivity). PCR/LAMP: Species-specific (limited availability).	Low awareness in non-endemic areas. - DEC unavailability for loiasis. - Poor treatment response in mansonellosis (Wolbachia-independent strains).	Loiasis and mansonellosis are underdiagnosed in non-endemic countries due to variable clinical presentation and low clinician awareness. Mansonellosis symptoms (e.g., abdominal pain) may warrant treatment despite historical perception as benign.
Guevara et al., 2022 [28]	Endemic regions: Mato Grosso, Brazil (tropical Amazon biome). Risk factors: Rural/agricultural exposure, male gender, comorbidities (hypertension, diabetes, leprosy)	Fungal: *Fonsecaea pedrosoi* (chromoblastomycosis). Other agents: *Cladophialophora carrionii*, *Phialophora verrucosa*	Chronic: mean duration: 8.6 years (range: 2–240 months). Skin signs: early lesions are asymptomatic; late-stage verrucous/cicatricial plaques. Utility: pathognomonic muriform cells on histopathology	Verrucous plaques (60% of cases). Cicatricial/scarring lesions (30%). Mixed forms (10%). Pruritus (50%), pain (40%), and foul odor (20%). Lower limbs (70%), upper limbs (30%)	Traumatic inoculation of melanized fungi via soil/plant exposure. Granulomatous inflammation with muriform cells ("copper pennies"). Immune evasion due to fungal melanin	DDx: Leprosy, paracoccidioidomycosis, squamous cell carcinoma. Misdiagnosis: 1 case initially treated as paracoccidioidomycosis for 10 years.	Microscopy: KOH mounts (80% sensitivity), histopathology (muriform cells). Culture: Potato dextrose agar. Molecular: ITS/β-tubulin sequencing, AFLP genotyping. -Antifungal MICs: Terbinafine (most active), voriconazole, and itraconazole.	Delayed diagnosis (mean 8.6 years). Treatment resistance: Long disease duration correlates with poor response. Comorbidities: Leprosy coinfection complicates management. Drug availability: Limited access to antifungals in rural areas.	Chromoblastomycosis disproportionately affects impoverished rural populations, with F. pedrosoi as the dominant agent in Brazil. Terbinafine, voriconazole, and itraconazole showed the lowest MICs, supporting their use as first-line therapies.
Hlela et al., 2008 [29]	Endemic: KwaZulu-Natal (KZN), South Africa. Other endemic areas: Caribbean, Japan, Brazil. Population: Predominantly African, low socioeconomic status. Co-infections: HIV (30% co-infection rate)	Pathogen: HTLV-1 (retrovirus) Infection: chronic dermatitis with bacterial superinfection (*S. aureus* 90%, *streptococci *68%)	Onset: early childhood (mean age 17 years; youngest 8 months). Diagnostic value: major criteria include scalp/axillae/ear eczema, nasal crusting, and a relapsing course. Prognostic: may precede HTLV-1 complications (HAM/TSP, ATLL)	Morphology: exudative crusted eczema + generalized papular rash (23.5%). Distribution: scalp (77.4%), retroauricular (71%), axillae (65%), paranasal (58%). Key signs: nasal discharge/crusting (48.4%), blepharoconjunctivitis (8.3%)	Immune dysregulation: elevated CD4/CD8, hypergammaglobulinemia. Viral mechanism: HTLV-1 alters T-cell function; bacterial superinfection maintains chronicity. Histopathology: superficial/deep perivascular dermatitis (38%); eosinophilia in HIV+ cases	Primary mimics: Seborrheic dermatitis (HIV-associated), atopic dermatitis, scabies Challenges: 12% lacked nasal crusting (revised diagnostic criteria needed)	Serology: HTLV-1 ELISA/Western blot (100% confirmation) Microbiology: Skin swabs (S. aureus/streptococci dominant) Histopathology: Lichenoid/interface dermatitis patterns Genotyping: Cosmopolitan subtype A (HTLV-Ia)	Underdiagnosis: Overlap with common eczemas Resource limits: Sample loss in public labs; no IgE/IgD testing Co-infections: HIV alters histology (eosinophilic infiltrates)	HAID is a marker for HTLV-1 and may precede ATLL/HAM/TSP. Revised criteria: Nasal crusting not mandatory (absent in 12%). Complications: Scabies (18.1%), corneal opacities (8.6%), HAM/TSP (6%)
Chêne et al., 2024 [30]	Metropolitan France (linked to travel from Africa, Asia, DROM-COM)	*Corynebacterium diphtheriae* (77%), *C. ulcerans* (23%); cutaneous diphtheria (toxigenic: 39%; non-toxigenic: 61%)	Early: pre-existing lesions (70%). Late: chronic non-healing ulcers (>2 weeks in 61.3%). Rarely suspected clinically (82% diagnosed post-microbiology)	Ulcerations (82%), fibrinous base (70.8%), erythematous-purplish edges (60.4%), crusted (33.9%), necrotic (16.1%). Lower limbs (86.9%), upper limbs (20.3%), head (10%)	Toxigenic: diphtheria toxin causes systemic complications. Non-toxigenic: biofilm formation, adhesion factors, co-infections with *S. aureus* (54.7%), *S. pyogenes* (49.1%)	Ecthyma (22.9%), impetigo (17.6%), leishmaniasis (14.7%), Buruli ulcer (5.9%); High misdiagnosis due to non-specific ulcers	PCR for tox gene (gold standard); Nasopharyngeal swabs (9.5% carriage); Serology underutilized	Underdiagnosis (17.5% ignored results); Low vaccination (36.6% unvaccinated); Poor contact tracing (42.6%); Inconsistent serotherapy (27.2% of toxigenic cases)	Chronic ulcers in travelers should prompt testing"; "Co-infections obscure diagnosis (88.9%); pseudomembranes are rare in non-toxigenic strains
Kalavacherla et al., 2022 [31]	Border region: US-Mexico endemic TB zone, high-risk population (homeless)	Disease: cutaneous DLBCL variant: necrotizing B-cell lymphoma non-infectious malignancy mimicking infection	Week 1: necrotic masses (TB suspicion). Week 2: ulcer progression. Week 3: biopsy confirms lymphoma. Skin signs initially misleading for TB	Bulky necrotic masses (10 cm neck, hemorrhagic axillary ulcers, cavitated back lesions, ecchymoses without trauma	Malignant B-cell infiltration → tissue necrosis. Angioinvasion → hemorrhage. Mass compression → vascular compromise. No true granulomatous pathology	1. Tuberculosis lymphadenitis (85% initial misdiagnosis). 2. Deep fungal infection. 3. Metastatic carcinoma. 4. Pyoderma gangrenosum, high risk due to partial RIPE response, endemic TB exposure	CT: necrotic masses with vascular compression. Histopathology: CD20+ B-cells. Microbiology: AFB/GMS negative, TB PCR negative. IHC: B-cell markers positive	Repeated nondiagnostic biopsies Empiric TB treatment delay Chemotherapy ineligibility Rapid fatal progression (4 weeks)	Cutaneous DLBCL perfectly mimicked TB in endemic zones. Necrosis + partial antibiotic response ≠ infection Vascular compression without fistulae suggests malignancy
Ribeiro et al., 2022 [32]	Not tropical-specific, occurs globally, higher risk in immunocompromised hosts	Pathogen: *Pseudomonas aeruginosa* (blood culture-confirmed). Infection type: septicemia with cutaneous manifestations (ecthyma gangrenosum)	Day 1-3: fever, vomiting, diarrhea. Day 3: papulonodular lesions and necrotic ulcers develop. Skin signs prompted antibiotic adjustment	Early: papulonodular lesions with erythematous halo. Late: central necrotic ulcers with hemorrhagic base. Distribution: legs and torso, no mucosal involvement	Bacterial invasion of dermal vessels → vasculitis. Toxin-mediated tissue necrosis. Neutropenia (1.5 x 10⁹/L) impairs containment. Hematogenous spread from the otorrhea focus	1. Other bacterial EG (Aeromonas, E. coli) 2. Fungal EG (Candida, Fusarium) 3. Vasculitic lesions 4. MRSA skin infections: High misdiagnosis risk in early stages	Blood cultures (confirmed *Pseudomonas*), wound cultures, CBC (neutropenia), CRP/procalcitonin (249 mg/L; 38.3 ng/mL), immunodeficiency workup	Delayed ulcer development masks diagnosis. initial antibiotics lacked Pseudomonas coverage Need for ICU care (vasopressors/ventilation) 5-week IV antibiotics required	EG lesions evolve from papules to necrotic ulcers—early recognition is critical. Neutropenia + hemorrhagic papules = empiric anti-pseudomonal coverage All EG cases warrant immunodeficiency screening."
Blattner et al., 2015 [33]	Endemic: West/Central Africa. Outbreak zones: Near the Ebola River basin. High-risk areas: forested regions with bat reservoirs	Pathogen: *Zaire ebolavirus *(Filoviridae). Infection type: viral hemorrhagic fever. Transmission: bodily fluids/bushmeat contact	Day 4-6: maculopapular rash onset (Figures 1-2). Day 8: dusky erythema generalization. Day 12-14: desquamation (palms/soles). Skin signs precede the hemorrhage phase	Early: centripetal macules (arms/legs → trunk). Mid-stage: dark-red papules (perifollicular). Late: hemorrhagic (petechiae/purpura). Ghost-like facial appearance. Mucosal: conjunctivitis/photophobia	Endothelial infection → microthrombi. Cytokine storm → vascular leakage. Coagulopathy (↓clotting factors). Multiorgan failure (liver/kidneys). Histology: dermal necrosis without inflammation	1. Other VHFs (Marburg/Lassa). 2. Measles/rubella. 3. Drug eruptions. 4. Meningococcemia. High misdiagnosis risk in the early maculopapular phase	Biopsy: formalin-fixed specimens (IHC). Serology: ELISA for antigens. Imaging: EM for viral particles. Lab: thrombocytopenia/DIC panels	Biosafety level 4 requirements PPE limitations in outbreaks No approved antivirals/vaccines High mortality (up to 90%)	The rash is a sentinel sign—it appears before hemorrhage. Perifollicular papules and fever = isolation trigger. Formalin-fixed skin samples enable safe diagnosis."
Belizario et al., 2016 [34]	Western Pacific/Southeast Asia High-risk areas: poor sanitation regions. Endemic zones: coastal/rural communities	Ectoparasites: *Sarcoptes scabiei* (scabies), *Pediculus humanus* (pediculosis). Helminths: *Ancylostoma braziliense* (CLM), *Strongyloides *(larva currens), *Schistosoma *spp. Protozoa: * Entamoeba histolytica*	Scabies: burrows appear 2-6 weeks post-exposure. LM: serpiginous tracks emerge 1-6 days after skin penetration. Larval currents: rapid migration (10 cm/hour). Cutaneous signs often precede systemic symptoms	Scabies: interdigital burrows, genital nodules. CLM: serpiginous erythematous tracks (Figure 1). Larva currens: urticarial linear rash. Schistosomiasis: zosteriform papules. Amoebiasis: indurated ulcers with pus	Scabies: type IV hypersensitivity to mites. CLM: larval proteases cause dermal tunneling. Filariasis: lymphatic obstruction → elephantiasis. Amoebiasis: *Trophozoite cytolysis* → necrosis	1. Bacterial pyodermas (scabies) 2. Contact dermatitis (CLM) 3. Fungal infections (schistosomiasis) 4. Malignancy (amoebiasis) High misdiagnosis in non-endemic areas	Microscopy: Scotch tape test (enterobiasis) Dermatoscopy: Burrow ink test (scabies) Biopsy: Granulomas (schistosomiasis) Serology: ICT cards (filariasis)	Social stigma delays care. Limited diagnostics in rural areas Drug resistance (ivermectin): Co-infections complicate treatment	CLM is the most common travel-associated dermatosis.Rapid larva migration distinguishes strongyloidiasis. Cutaneous schistosomiasis often indicates chronic infection
Marques, 2013 [35]	Endemic: Brazil, Colombia, Venezuela. Ecology: soil, armadillo burrows. Risk: rural workers, males 30-50, HIV+	Fungus: *P. brasiliensis *complex. Transmission: airborne spores. Forms: acute (systemic), chronic (mucocutaneous)	Early: asymptomatic. Chronic: skin/mucosal lesions (diagnostic). HIV+: skin 66.7%, fewer mucosal	Mucosal: Mulberry ulcers. Cutaneous: verrucous plaques, sarcoid-like leprosy mimic. Lympho: abscessed nodes	Immune: Th1 granulomas. Fungal: thermal dimorphism. HIV: false-negative serology	TB, leprosy, and SCC HIV confound presentation	Histo (gold standard), serology (ELISA), PCR (emerging), culture (37.5% sense)	Lab delays in rural areas HIV alters presentation. Long treatment (6-24 mo)	Skin lesions trigger diagnosis" 75% relapse if non-adherent
Ferraresso et al., 2018 [36]	Endemic areas: Argentina (Córdoba), Latin America. Non-endemic spread: global via migration/transplants. Risk factors: Ural exposure, immunosuppression	Pathogen: *Trypanosoma cruzi* (protozoan). Transmission: vector (Triatoma infestans), transplant, transfusion. Reactivation trigger: immunosuppression (tacrolimus/mycophenolate)	Latent phase: asymptomatic (indeterminate/chronic). Reactivation: 3-month post-transplant. Key sign: cutaneous lesions = early reactivation marker	Chagasic hypodermitis: painful erythematous plaques (thighs/glutei/arm). Acute phase signs: "Chagoma" (inoculation nodule), "trypanosomides" (morbilliform rash)	Immune evasion: intracellular amastigotes (heart/brain/skin). Reactivation: loss of immune-parasite balance. Tissue tropism: dermal/hypodermal infiltration	High Misdiagnosis Risk: Cellulitis, erythema nodosum, drug rash, HIV/transplant confounders, and Atypical presentations	Gold standard: PCR (blood/tissue: 76,785 eq.p/mL). Histopathology: Giemsa-stained amastigotes in macrophages. Serology: ELISA/hemagglutination (pre-transplant screening)	Monitoring Gaps: weekly PCR not performed Treatment Barriers: metronidazole toxicity (leukopenia/hepatotoxicity) System Failures: Disconnected follow-up	Cutaneous lesions are a sentinel sign of reactivation.20-40% reactivation risk in transplants Global awareness needed even in non-endemic areas
Diaz, James H., et al., 2016 [37]	Endemic areas: Southeast Asia (Thailand, Japan), Latin America (Mexico). Emerging regions: Central/South America. Risk factors: consumption of raw/undercooked fish, shrimp, frog, or chicken	Pathogen: *Gnathostoma spinigerum *(helminth). Transmission: ingestion of L3 larvae in food. Forms: cutaneous (80%), visceral, CNS (rare)	Incubation: weeks to years. Key sign: migratory swellings (diagnostic hallmark). Chronicity: symptoms persist for months/years without treatment	Cutaneous: intermittent, pruritic, migratory swellings (3-6 cm). Visceral: eosinophilic gastritis, abdominal pain. CNS: radiculopathy, eosinophilic meningitis	Larval migration: L3 larvae wander through tissues, provoking inflammation. Eosinophilia: modest (0.4-4.4 x 10⁹/L) human dead-end Host: larvae cannot mature	High Misdiagnosis Risk: - Cellulitis - Allergic reactions - Loa loa (Calabar swellings) Delayed Diagnosis: Median 12 months in study	Gold standard: immunoblot (24-kDa band). Imaging: MRI for muscle lesions (e.g., vastus lateralis). Histopathology: eosinophilic infiltrates (gastric/skin biopsies)	Diagnostic Delays: Lack of clinician familiarity in non-endemic areas Treatment Failures: 3/16 patients required a second albendazole course.Dietary History: Often unreliable	Migratory swellings are pathognomonic but often overlooked. Albendazole efficacy is >90%. Global travel = rising incidence in non-endemic regions
Buell et al., 2019 [38]	Endemic areas: Central Africa (Cameroon, Gabon, DRC). High-risk: forested regions (≥20% microfilaremia prevalence). Global spread: imported cases in non-endemic countries	Pathogen:* Loa loa *(filarial nematode). Transmission: Chrysops fly bites. Forms: cutaneous (Calabar swellings), ocular (eyeworm), visceral/systemic	Classic signs: transient subcutaneous swellings (Calabar), conjunctival migration (eyeworm). Atypical onset: weeks to years post-exposure. Key utility: swellings signal active infection	Cutaneous: pruritic, migratory swellings (3-10 cm). Ocular: adult worm in conjunctiva/anterior chamber. Visceral: organ-specific inflammation (e.g., pleural effusion, endomyocardial fibrosis)	Immune response: eosinophilic inflammation (Th2-driven). Microfilarial burden: high densities (≥8000 mf/mL) linked to severe disease. Tissue damage: migrating larvae provoke granulomas/fibrosis	High Misdiagnosis Risk: cellulitis/allergic reactions Onchocerciasis (ocular) Lymphatic filariasis Delayed Diagnosis: Median 12 months in non-endemic settings	Gold standard: microscopy (mf in blood/CSF). Serology: immunoblot (24-kDa band). Imaging: MRI/CT for visceral involvement (e.g., cardiac fibrosis). PCR: confirmatory in atypical cases	Diagnostic Gaps: Limited access to specialized tests in endemic areas Treatment Risks:Ivermectin is contraindicated in high mf densities (SAEs). Co-Infections: Mansonella perstans complicates management	47% of cases present atypically, affecting vital organs." High mf densities (≥8000/mL) increase odds of atypical manifestations. 9-fold Loiasis remains absent from WHO. NTD priority list despite mortality links
Friedman & Schwartz, 2019 [39]	Global (India, Brazil, Africa, etc.)	*Emergomyces *spp. (disseminated mycosis); *Blastomyces helicus* (atypical blastomycosis)	Skin lesions are common in disseminated emergomycosis (HIV patients); late-stage diagnosis is frequent	Ulcerative, nodular, or papular lesions; often widespread in emergomycosis	Immune evasion in HIV; thermal dimorphism, and environmental exposure	TB (misdiagnosed in 25% of emergomycosis cases); other endemic mycoses.	Blood cultures, histopathology, and molecular sequencing.	Low clinical suspicion in non-endemic areas; delayed diagnostics.	"Half of emergomycosis cases were diagnosed post-mortem.
Silva-Ramos et al., 2023 [40]	Endemic regions: Amazon rainforest (Brazil, Peru, Venezuela, Trinidad and Tobago, Haiti). Vector: primarily *Haemagogus janthinomys* (potential urban adaptation by *Aedes *and *Culex* spp.). Seasonality: linked to tropical rainy seasons	Mayaro virus (MAYV): single-stranded RNA virus (Alphavirus genus, Togaviridae family)	Age: young adults (39%) and middle-aged adults (25.8%). Most affected sex: males (56.1%) > females (43.9%). High-risk groups: forest workers, farmers, ecotourists	Most common symptoms: fever (90.6%), arthralgia (63.7%), headache (61.5%), myalgia (50%), retro-orbital pain (47.4%). Rash: maculopapular/macular (23.9%). Complications: chronic arthralgia (rare, 4.3%)	Normal parameters: 82.8% of cases. Abnormalities (17.2%): leukocytosis (39.1%), leukopenia (34.8%), thrombocytopenia (21.7%), elevated ALT (21.7%)	Methods: RT-PCR (acute phase), ELISA/IFA (serology, cross-reactivity risk), PRNT (confirmation) Challenges: Misdiagnosis with Chikungunya due to overlapping symptoms	Therapy: Supportive (hydration, anti-inflammatories; 88.9%) Hospitalization: Rare (5%) Outcome: Full recovery in all cases; no fatalities reported	Autochthonous Cases: 96.6% (Peru [54.9%], Brazil [38.1%]) Imported Cases: 3.4% (Europeans traveling to Amazon regions) Emerging Threat: Potential urbanization via Aedes/Culex vectors	Mayaro fever appears to be a mild, self-limited disease... but its true pathogenic potential remains underestimated.
Ta-Tang et al., 2018 [41]	Endemic regions: Sub-Saharan Africa (*M. persians*, *M. streptocerca*), Latin America/Caribbean (*M. ozzardi*). Vector: Culicoides midges (all species), Simulium blackflies (*M. ozzardi*). Co-circulating pathogens: Malaria, onchocerciasis, and lymphatic filariasis	Filarial nematodes (*Mansonella spp.*). Infection type: chronic, asymptomatic in >90% of cases. Microfilariae circulate in blood (*M. persians, M. ozzardi*) or skin (*M. streptocerca*)	Skin signs: rare; if present, develop months/years post-infection. Utility: low specificity; *M. oozzardi *is linked to corneal lesions (Brazil), and *M. treptocerca* to papular dermatitis (Africa)	*M. streptocerca*: pruritic papules/hyperpigmented macules (shoulders/thorax). *M. Ozzardi:* corneal opacity (Brazil). *M. persians*: non-specific Calabar-like swellings (rare).	Microfilariae evade immune detection (chronicity). *Wolbachia *endosymbionts (*M. persians/ozzardi*) may modulate inflammation. No direct tissue destruction; symptoms are likely immune-mediated	High Misdiagnosis Risk: *M. ozzardi *vs. *Onchocerca volvulus *(skin snips). M. perstans vs. Loa loa (blood smears). DDx: Onchocerciasis, loiasis, drug reactions.	Microscopy: Gold standard (Giemsa-stained blood/skin snips). Molecular: PCR (rDNA ITS1) to differentiate species. -Serology: Limited due to cross-reactivity.	Asymptomatic cases obscure the burden. Morphological confusion with other filariae. No WHO-endorsed treatment guidelines. Resource limitations in endemic areas.	Mansonellosis is among the most neglected tropical diseases despite affecting more than 100 million people. CR-based tools are critical to distinguish sympatric filarial species in co-endemic areas."
Rezza et al., 2017 [42]	Endemic areas: Sub-Saharan Africa (Uganda, Kenya, Tanzania, Cameroon, Senegal). Vectors: *Anopheles funestus *and *A. gambiae* (malaria vectors). Co-circulating pathogens: malaria, chikungunya (CHIKV)	Virus: O'nyong-nyong virus (ONNV), an Alphavirus (Togaviridae). Transmission: mosquito-borne (primarily *Anopheles* spp.). Zoonotic potential: unknown reservoir; suspected sylvatic cycle	Onset: 8-day incubation. Skin signs: a maculopapular rash (itchy) appears early (days 1-3), concurrent with fever. Utility: supports clinical diagnosis but overlaps with CHIKV (lymphadenopathy is a key differentiator)	Generalized maculopapular rash (often pruritic). Unlike CHIKV, it is associated with posterior cervical lymphadenopathy (60-80% of cases). No hemorrhagic manifestations (rare bleeding gums/nosebleeds)	Viral replication in joints/muscles → severe arthralgia. immune-mediated symptoms (cross-reactive antibodies with CCHIKV). Nopheles midgut/innate immunity modulates transmission (unique among alphaviruses)	High Misdiagnosis Risk: CHIKV (similar arthralgia/rash; lacks lymphadenopathy). dengue, measles, and rubella. Key Differentiator: Cervical lymphadenitis + Anopheles exposure.	Serology: IgM ELISA (cross-reacts with CHIKV; confirm with PRNT). RT-PCR: Viral RNA in acute-phase blood.Viral Isolation: Rare (requires BSL-3 facilities).	Limited surveillance in endemic areas. Serologic cross-reactivity with CCHIKV and commercial diagnostics/vaccines. symptomatic overlap with malaria co-infections.	ONNV is uniquely transmitted by Anopheles mosquitoes, complicating vector control. CHIKV vaccines may cross-protect due to antigenic similarity.

Reassessing the diagnostic utility of dermatologic signs

Early vs. Late Indicators

The fact that the skin symptoms act as precursors in treating tropical infections is partly correct, yet mostly pathogen-dependent. There are known infections where rashes appear on the skin 24-48 hours after the beginning of the symptoms, which include dengue and Zika virus, which is why such infections could be useful in terms of early clinical suspicion and triage [16,39]. Exanthemas, fever, and myalgia, as well as conjunctivitis, tend to accompany each other in these viral infections to give clear syndromic disturbances. Nevertheless, such a tendency is not present everywhere. Leishmaniasis and chromoblastomycosis are dermatologic conditions that present themselves in a delayed manner. The parasite takes several weeks after a sandfly bite to cause the typical leishmaniasis ulcers as the parasite travels extensively around the dermis system [17]; thus, the time it takes for chromoblastomycosis lesions to form, months to years after a trauma, makes it less relevant as an acute diagnosis method [28]. Thus, dermatologic manifestations of tropical infections vary and can be considered on two sides: early-stage indicators and late-stage findings, and they can hardly be used as unconditionally early signs.

**Table 2:** Summary table of early vs. late skin indicators *M*. *ulcerans*: *Mycobacterium ulcerans; *DLBCL: diffuse large B-cell lymphoma; EG: ecthyma gangrenosum; TB: tuberculosis; PCR: polymerase chain reaction

Pathogen/infection	Early skin signs	Late skin signs	Clinical utility
Dengue virus	24-48 hours: flushing erythema, morbilliform rash	3-6 days: maculopapular rash, petechiae, “white islands in red sea”	Early rash aids timely differentiation from chikungunya/measles; lab confirmation often delayed
Chikungunya virus	1-7 days: maculopapular rash (trunk/limbs/face), aphthous ulcers	1-2 weeks: pigmentary changes, desquamation	Early recognition prevents misdiagnosis; groin ulcers, male-specific and pathognomonic
Zika virus	24-48 hours: pruritic maculopapular rash, conjunctivitis	3-10 days: reticular patterns, psoriasiform plaques	Early rash pathognomonic; early NAT <7 days required for confirmation
Cutaneous leishmaniasis	2-8 weeks: papules/nodules	Ulcers with raised borders	Early detection is critical in resource-limited settings
Onchocerciasis	Chronic: hypopigmented macules (leopard skin), nodules	Long-term: nodules, severe pruritus	Early recognition is essential where lab resources are scarce
Cutaneous anthrax	1-7 days: painless papule	24-48 hours: vesicle → black eschar	Early papule is critical to avoid delayed antibiotic therapy
Scrub typhus	3-10 days: eschar with erythema	Complications: necrosis, atypical ulcers	Eschar pathognomonic; PCR from eschar is highly sensitive
Buruli ulcer (*M. ulcerans*)	Weeks-months: firm, nontender nodule	Undermined necrotic ulcers, satellite lesions	Early-stage diagnosis prevents disability
Cutaneous DLBCL	Week 1: necrotic mass (misleading for TB)	Week 3: ulcerative progression, biopsy confirms malignancy	Misleading early signs; rapid biopsy essential for diagnosis
Pseudomonas EG	Day 3: papulonodular lesions	Day 5: necrotic ulcers	Early recognition is critical in immunocompromised; it guides empiric anti-pseudomonal therapy
Ebola virus	Day 4-6: centripetal macules	Day 12-14: desquamation, hemorrhage	Early rash triggers isolation; precedes hemorrhagic phase

Skin as a Window

The traditional doctrine that the skin is the reflection of the inner activities of the system is seductive enough but is unreliable in tropical medicine. In contrast to the autoimmune or metabolic diseases, in which skin alterations may be based on fixed systemic pathways, tropical infections embrace a large variety of possible pathogens and dysfunctional mechanisms and manifestations. The skin signs become mixed in tropical settings, where polyparasitism, chronic malnutrition, poor sanitation, and inappropriate use of medication are prevalent. Such skin diseases as scabies and cutaneous larva migrans, also, are often confused with eczema or allergic dermatitis, or even remain undiagnosed without dermoscopic equipment or microscopes [24]. Drug hypersensitivity reactions or frequent viral diseases may be mimicked by a viral exanthem, either in the form of Chikungunya or in the form of Zika [15]. This leads to a situation in the diagnostic environment in which clues of a dermatologic nature may either be misleading or unnoticed. Hence, even though the skin can still be used as a window, due to intersecting symptoms, co-infection, and constraints in healthcare, the window is usually obscured.

Specificity and Misdiagnosis Risks

The low specificity and the high incidence of the misdiagnosis of the cutaneous signs can be considered as one of the key issues in clinical dermatology in tropical medicine. Incidentally, these diseases create a broad confusion, such as in the case of the Chikungunya maculopapular rash, where ranking among the highest misdiagnoses in outbreak situations is 37 percent due to its likelihood to be confused with dengue or drug eruptions [22]. In the same way, scrub typhus eschar may be a pathognomonic sign and is often mistaken for anthrax, syphilitic chancres, or herpetic lesions, except that lesions on the genital areas or axilla may be mistaken for scrub typhus [19]. Leptospirosis, which is diagnosed with conjunctival suffusion and petechiae, is commonly mistaken for viral hemorrhagic fever (VHF) or dengue because of the coincidence of systemic signs and the non-availability of diagnostic methods [26]. Such pitfalls in diagnosis prolong the time of treatment and make the identification of an outbreak more difficult, and introduce the possibilities of inappropriate antibiotics or unwarranted isolation.

Skin Tropism and Pathogen-Host Interaction

When it comes to the skin tropism of a pathogen, immunopathology, and host response, the probability that a tropical infection will present with dermatologic manifestations is frequently observed. In others, e.g., onchocerciasis and *Loa loa*, the skin serves as a reservoir and battlefield so that strong inflammatory reactions to the microfilariae result in chronic itching and the alteration of pigmentation (to give a leopard skin appearance) plus nodules [18]. The cutaneous involvement of the Zika virus is manifested via the direct cell targeting, i.e., the envelope protein of the virus binds to AXL receptors of the keratinocytes and dendritic cells and binds to them, causing the rash and local inflammation [16]. The pathogen of Leishmaniasis infects dermal macrophages, and this results in granulomatous inflammation and ulceration of the skin, whereas immune-mediated vasculitis plays a role in causing rash, purpura, and pigmentary changes in chikungunya. Such differences point to the reason behind the existence of some infections being characterized by apparent skin manifestations and others that are not. Other diseases, such as melioidosis or Hantavirus, on the contrary, seldom leave cutaneous hints unless the disease is either complicated by the formation of abscesses or has some hemorrhages. So, there is the biology of the pathogen as well as the immune behavior of the host, which produces the presence or absence of dermatologic manifestations.

A syndromic framework: improving diagnostic reasoning

A syndromic system relies on sets of signs and symptoms, which are mainly the clinical signs and symptoms, specifically dermatologic signs and symptoms, to diagnose in the absence of laboratory tests, in order to base the diagnosis. The unique position of the skin signs is that they could facilitate early clinical suspicion because they are visible and accessible. As an example, a combination of symptoms such as fever, rash, and conjunctivitis is one of the distinctive signs of the Zika virus, especially in its outbreak areas [25]. In the same manner, another condition, i.e., the presence of ulcer + lymphadenopathy, may point to either yaws or cutaneous Leishmaniasis, considering such factors as the geographical area and history of exposure [26]. Nevertheless, the validity of these schemes in low-resource or hyperendemic environments is not routinely demonstrated, and they often remain silent on the dermatologic subtleties, including pigmentation of the lesion, location of the eschar, or alteration of presentation according to patient age. As an example, the chances of developing scrub typhus eschars at trophic sites are higher on the infra-umbilical region in adults of male gender, but the chance of this region being covered by dark skin may be ignored [24]. In a bid to enhance usability and accuracy, the adoption of AI-enabled image recognition platforms, as well as teledermatology solutions in the screening of tropical diseases, has become appealing. Although they seem to be promising, such technologies require high-quality and geographically representative image datasets and training data on skin tones [22]. In the absence of such a base, there exists the possibility of AI and remote diagnostic tools being used as a means of reinforcing pre-existing diagnostic biases. Syndromic frameworks serve as practical tools for the rapid recognition of tropical infections, especially in resource-limited settings. They allow healthcare workers to identify likely infections based on presenting signs and symptoms, facilitating timely management. However, these frameworks cannot replace confirmatory laboratory testing, which is essential in cases of co-infection or drug hypersensitivity reactions. Clinical presentations may also be atypical in immunosuppressed patients, making careful interpretation critical. Despite these limitations, syndromic approaches can enhance diagnostic efficiency and support early intervention. They are particularly valuable when integrated into clinical decision support tools and training modules. By guiding initial assessment, these frameworks improve healthcare worker preparedness and patient outcomes. 

Pathogen-specific dermatologic profiles: synthesis with critical insights

In immunosuppressed patients, particularly those with HIV, dermatologic manifestations of tropical infections may present atypically. Lesions can be subtle, rapidly progressive, or lack classic features, and may mimic other conditions such as malignancy or adverse drug reactions. These atypical presentations highlight the importance of considering a patient’s immune status and maintaining a high degree of clinical suspicion when evaluating skin findings in tropical settings.

Viral Infections

The clinically significant problem with viral tropic infection is the morphological similarity of the exanthems. Dengue, Zika, chikungunya, and Mayaro are commonly found with maculopapular rashes that are either non-pruritic or have mild pruritus and can be observed on the trunk and the limbs. The pattern, distribution, and duration of such rashes are often indistinguishable, and therefore, the clinical distinction between different rashes is quite infeasible without laboratory verification [18,22]. Where these viruses co-circulate, the diagnostic ambiguity is aggravated, and therefore, there is a high rate of clinical misclassification. As an example, the blanching, reticular maculopapular rash that could be mostly accompanied by non-purulent conjunctivitis with slight itching can be the sign of Zika virus infection. Even though this cluster of results may help in the syndromic diagnosis, they do not have specificity in endemic areas. As a matter of fact, 70% of the Zika cases are underdiagnosed in case there is a co-infection with chikungunya and dengue [23]. In the meantime, monkeypox shows a distinct vesiculopustular rash that develops by stages of macules, papules, vesicles, and crusts; rather commonly, it affects the face, palms, and soles. Nevertheless, when it occurs in non-endemic regions, they often incorrectly diagnose it as varicella or herpes simplex, or secondary syphilis, because they are not familiar with it. Moreover, it shares certain similarities [28]. Ebola virus disease may have an impressive but underreported productive perifollicular, so-called ghost-like rash, which may delay hemorrhagic signs by days. It is a rather non-obvious but distinct determinant that provokes early confinement in conditions of outbreaks [33]. This notwithstanding, these findings are not mostly included in the diagnosis of outbreaks or surveillance. Altogether, in viral infections, there is an inadequacy in using the rash morphology as the primary parameter alone because in co-endemic areas, its use is not proper. To be successful in syndromic diagnosis, it is necessary to focus on accompanying symptoms of a given diagnosis (arthralgia in the case of chikungunya, retro-orbital pain in the dengue case, and conjunctivitis in the case of Zika) that can be used to increase diagnostic specificity without the use of laboratory tests [34,35].

Bacterial Infections

The skin lesions in bacterial infections in tropical areas tend to be more localized and progressive than viral exanthems, and such lesions, when identified early, could be more specific. As an example, black necrotic eschar with erythema lesions around and the presence of regional lymphadenopathy are classical manifestations of scrub typhus. The location, though, depends upon geographical as well as age aspects; it may be located in the groin, axilla, or inframammary areas, which can be unnoticed, especially in individuals who have darker skin color [36]. The strain variability determines the occurrence and the presence of eschars as well, without being recognized in the non-epidemic regions. There are a few nonspecific dermatologic changes in leptospirosis. The only relatively definite quality, conjunctival suffusion, is commonly missed even in darker-skinned patients or in resource-deprived jurisdictions without the availability of a slit lamp. Petechiae and purpura, in their occurrence, are normally mistakenly considered to be dengue or viral hemorrhagic fevers [37,38]. In the meantime, Buruli ulcer is a disease whose causative agent is *Mycobacterium ulcerans*, and it normally starts as a firm subcutaneous nodule, which then develops into an undermined, painless ulcer. The necrotic base and indolent nature of the ulcer usually resemble the fungal infections or neoplasms, or deep-seated bacterial abscesses, and it is not that simple to clinically identify the ulcer [39]. On the whole, even in spite of the clinical usefulness of patterns with the localization and development of lesions, bacterial dermatoses (anthrax, yaws, and Buruli ulcer) rely most strongly on early detection and local recognition. Otherwise, diagnosis slippage or wrongful diagnosis is still possible, particularly in non-specialist units.

Parasitic Infections

There is a two-part involvement of the skin in parasitic diseases as the portal of entry and the place where the primary action of the immune response occurs. As an example, cutaneous Leishmaniasis is transmitted by the bite of a sandfly, resulting in a skin ulcer localized in the area of infection weeks later, which can be surrounded by induration and even satellite infection [40]. Typically, these ulcers may be mistaken for bacterial abscesses or fungal infections at the initial phase. Cutaneous larva migrans is the result of the migration of helminth larvae under the skin, forming serpiginous, itchy lesions. Most often, it can be observed on the feet or on the buttocks of people who walk around without shoes. In the textbooks, such lesions are unique, but in practice, they are often misinterpreted as fungal tinea or dermatitis because of the fact that they change and shift in morphology. The filarial parasite *Loa loa* leads to Calabar swellings, which refer to migratory, painless swellings of the limbs, which may be similar to angioedema or cellulitis and which repeatedly result in the use of inappropriate antibiotics [18]. Onchocerciasis gives the most classical dermatologic aspects of the parasite. Repetitive inflammatory reactions of the microfilariae produce leopard skin pigmentation, nodules, and chronic itching in the affected patients in hyperendemic regions. However, these signs, being unique, are overlooked and, at best, unrecognized beyond the endemic areas or in the low-resource environments with scarce training. The fact that there is no uniform classification of lesions and regional dermatologic atlases is one of the significant impediments to the recognition and case reporting in various endemic areas [30,42].

Fungal Infections

The most common fungal infections of the tropics can be described as soil-related, chronic, and mostly unattended. The chromoblastomycosis and mycetoma diseases are also contracted through traumatic inoculation and are common among people who work on farms. These infections manifest themselves as slow-healing verrucous plaques or nodules or sinuses that may be confused with skin cancer, tuberculosis, or deep bacterial abscesses. A case in point is the mycetoma, which is occasioned by the actinomycetes or the fungus and manifests itself with the appearance of multiple sinus tracts carrying grains, usually on the foot [31,32]. Under climate change, these infections are becoming prevalent outside their classical endemic territories, meaning that they are spreading across the geographic climes. Areas that were hitherto characterized by dry or temperate climates are currently reporting intermittent cases of chromoblastomycosis as well as sporotrichosis, mostly in the absence of immunosuppressed people or migrant workforces [28]. This growth raises awareness and readiness in the coastal areas that have never been affected. A proper diagnosis of such infections would necessitate the availability of histopathology (e.g., excretion of muriform bodies in the case of chromoblastomycosis), dermoscopy, or culture, not normally available in those rural tropical clinics. Therefore, dermatoses caused by fungi are wrongly diagnosed or not informed until the last stages. The gaps need to be filled urgently by capacity building in the fields of skin biopsy, microscopy, and fungal diagnostics in endemic regions [26].

Geospatial critique: dermatologic infections in a changing world

The spatial distribution of the viruses leading to dermatologic infections due to vectors has expanded owing to climate change. Disease-causing agents such as *Leishmania *that were at one time predominant in the arid parts of the equator are now found in Southern Europe as a result of enhanced sandfly habitats [20]. These different climate trends have resulted in new epidemics in other non-endemic areas, surprising even the healthcare facilities. Urbanization worsens the condition by congregating vulnerable groups in the peri-urban slums, where they do not prevent the circulation of tropical pathogens such as Buruli ulcer or cutaneous larva migrans because of inadequate sanitation and the lack of healthcare facilities. This causes a rise in the difficulty of diagnosing in such settings due to the high background prevalence of overlapping dermatoses and a lack of specialists [19,21]. Owing to these changing issues, the dermatology and infectious disease curricula are stagnant in most areas. There is still a high likelihood that clinicians receive the training on the most common Western skin conditions, such as acne, psoriasis, and atopic dermatitis, rather than learn tropical dermatoses and the growing condition with pathogen-related rashes [17]. Such a mismatch between the global epidemiology of diseases and healthcare education presents a possible threat of increasing the time spent on diagnosing new infections and responding to imported ones.

Challenges in clinical practice

Incorrect diagnosis of tropical skin infections is still a problem of great concern in the clinical management of tropical skin infections. There is constant confusion that arises between viral exanthems and drug eruptions, and common inflammatory dermatoses because they overlap. As an example, rashes of Zika or chikungunya are sometimes misunderstood as hypersensitivity to drugs, and skin manifestations of scabies or cutaneous larva migrans are conflated with eczema [15]. To worsen this, most of the tropical nations have a shortage of specialized dermatologists, and therefore, general physicians, who are not trained in dermatology, are supposed to treat skin diseases. This gap in knowledge leads to the development of heuristics in diagnosis, which are mostly inaccurate, especially when these cases are presented in an unusual manner [21]. The other big business hurdle is confirmatory testing. Skills like skin biopsies, PCR assays, or even histopathology are not within the reach of a rural or underresourced clinic, and as a result, providers are left with little to do but to use visual inspection as their sole method, which is always subjective and incorrect [27]. In case of such diseases as Buruli ulcer or anthrax, it may postpone the treatment and even trigger unnecessary surgeries. In high-income countries, subtle skin manifestations or signs that are unfamiliar in high-income countries are usually ignored in a travel clinic setting, more so when the returning traveler has a darker skin color, where the lesions are not visible or are differently manifested. Such lapses may lead to failure in isolation strategies, reporting of an outbreak, and even detection of cases of imported diseases such as monkeypox or leishmaniasis [31,32]. Changing geographies can significantly impact clinical diagnostic reasoning, as clinicians practicing in non-endemic regions may misinterpret or completely overlook tropical skin manifestations. Familiarity with common local conditions shapes pattern recognition, so when a patient presents with signs typically seen in tropical areas, providers in temperate regions may fail to identify them promptly. This can lead to delayed diagnosis, inappropriate treatment, or unnecessary investigations. Incorporating awareness of geographic context into training and decision-support tools helps clinicians anticipate atypical presentations. Such knowledge is particularly important for returning travelers, migrants, or immunosuppressed patients who may present with unusual or subtle lesions. By considering geographic exposure, clinicians can improve diagnostic accuracy and optimize patient outcomes. Overall, integrating geographic context strengthens the practical utility of syndromic frameworks in diverse clinical settings.

Knowledge gaps and research directions

One of the most pressing gaps is the underreporting of skin signs in both clinical case reports and outbreak surveillance. Dermatologic findings are often absent or inadequately documented, limiting their utility for clinicians and public health teams attempting to track emerging trends. For example, reports from Ebola outbreaks frequently described cutaneous hemorrhage but omitted early indicators such as perifollicular rashes, which could serve as key predictors [33]. Additionally, there is a scarcity of high-resolution, annotated clinical images from tropical regions. Existing image banks are largely Western-centric, underrepresent darker skin tones, and fail to capture unusual infections, limiting the applicability of AI-based diagnostic systems and the scalability of teledermatology programs [32]. Interobserver variability further complicates diagnosis, as clinicians with different training or from different geographic regions may classify identical lesions differently; yet, few studies have formally evaluated the reliability of dermatologic assessments in tropical settings [28]. To address these gaps, priority should be given to establishing multicentric registries of tropical dermatologic diseases that integrate standardized clinical photography, microbiologic confirmation, geolocation, and patient demographics. Such repositories would support lesion classification, enhance AI algorithm training, and enable teledermatology programs. Coupled with international consensus on reporting standards and the application of implementation science, these efforts could strengthen disease surveillance, improve diagnostic consistency, and accelerate translational impact in global tropical dermatology [20].

Educational and Policy Implications

Redesigning of the medical and public health education: There is a burning need for a redesign of this medical and public health education so as to have comprehensive training in tropical dermatology. Today, the skin modules in infectious disease, emergency medicine, and global health programs are usually superficial or nonexistent. The interdisciplinary training, especially in the process of diagnosing tropical and neglected skin diseases, may be highly effective in implementing frontline diagnosis precision [39]. The second significant proposal to its policy includes creating open-source dermatoscopy image libraries with a variety of skin tones, as well as the ones of the region-specific infections. These libraries could be beneficial both to clinicians in terms of visual recognition and to the training of AI models in terms of mobile app creation and e-learning solutions [28]. Lastly, the global health preparedness strategies need to include dermatologic surveillance, particularly in refugee camps, disaster areas, and migrant health schemes. Skin manifestations remain the first visible manifestation of a possible indicator of new agents in such environments, as an outbreak of infectious diseases can be frequent, and their systemic symptoms are delayed or covered [18,33]. The enhancement of the visual diagnostic parameters within these areas may be the key to the prevention and control of the outbreak and decrease in morbidity.

The results of this review confirm the key importance of dermatologic manifestations as initial diagnostic criteria in tropical infections, being consistent with the past studies pointing to the clinical importance of cutaneous hints. Friedman and Schwartz addressed the usefulness of rash morphology and timing when it comes to distinguishing between such arboviral diseases as dengue, Zika, and chikungunya [39]. Despite the fact that the viral infections sometimes may have overlapping maculopapular dermatitis, minor differences, like the reticular blanching rash and non-purulent conjunctivitis of Zika, can provide some direction in the interpretation of its inclusion into the specific epidemiological setting. Nevertheless, in line with the remarks provided by Monsel and Caumes, this assessment is revealed to have a narrow reliability in diagnosing skin signs due to high non-specificity rates and inconsistency in the communication method [43]. Most skin lesions, especially those seen in bacterial infections, such as rickettsioses, may be different in appearance, and in low-resource settings that do not involve dermatology care, skin lesions may be underreported. This issue is reflected by the difficulties outlined by Crump et al., according to whom a number of rickettsial infections might have been underdiagnosed because of incomplete identification of dermatologic manifestations [44].

Conversely, cutaneous leishmaniasis and mycetoma are illnesses that host fairly unique dermatologic presenting features that are documented to a considerable degree in the clinical and epidemiologic studies. The disease, cutaneous leishmaniasis, generally contains chronic ulcerative plaques that usually help in early presumptive diagnosis of the disease in the endemic regions, as indicated in reports by Reithinger et al. [45]. We affirm these results; however, histopathologic or parasitologic confirmation is also necessary because of the appearance resemblance of these lesions to deep fungal infection or chronic wounds of other causes. Notably, such a review illustrates how uncommon dermatology-specific reporting is in outbreaks, which has already been mentioned in a meta-analysis conducted by Smith et al., as only 19.2% of reported infectious disease outbreaks contained a description of dermatologic findings and 23.9% any image of dermatologic abnormalities [46]. Most of the primary sources we used in our synthesis employed either anecdotal evidence or descriptions made by laypeople and, as such, were inconsistent in the terminology of lesions (calling them a rash, spots, or ulcers). This imprecision not only affects clinical identification but also interferes with the process of creating digital tools or machine learning applications of dermatologic triage during the outbreak of an infectious disease.

On top of this is the fact that the rise in climate change and international travel has led to the spread of the geography of the vectors, which has resulted in additional geographies that are being infected on a regular basis. According to the elucidation of Carlson et al., the clinicians in non-tropical regions will have to deal with the tropical intrusion into the skin that they have never seen before [47]. We tend to agree with this review, which suggests increasing cross-specialty training in the field between dermatologists and infectious disease professionals. This is particularly important to frontline healthcare providers in such places as travel clinics or refugee camps, for patients in underserved tropical areas where dermatologic manifestations are the only initial indication of disease. In general, the review backs up the prior requests to adopt better clinical algorithms, which include the involvement of dermatologic manifestations, exposure to the geographical region, along with the incubation period, and systemic symptoms and signs. We believe that a visual diagnostic atlas, together with real-world images and standardized terminologies, can be built on the basis of such frameworks as the one suggested by Molkara et al. [48]. Such instruments would be of great benefit to make the syndromic surveillance much more profound, faster to detect outbreaks, and better for patient outcomes by earlier treatment, especially in low-resource settings where skin is both a visible and priceless diagnostic layer. Improving dermatologic training in tropical medicine is critical to reducing diagnostic delays and mismanagement. Specific strategies include adding dermatologic modules to primary care training, promoting dermatology-infectious disease liaison roles, and implementing policy incentives to support specialized training in endemic areas. By directly linking educational gaps to clinical outcomes, these measures can enhance clinicians’ ability to recognize and manage tropical skin diseases effectively. Additionally, fostering interdisciplinary collaboration and integrating these initiatives into broader public health programs can strengthen outbreak response, disease surveillance, and overall patient care in tropical settings.

## Conclusions

Evaluation of skin findings indicates that these can be a significant source of diagnostic information, but in a contextually sensitive way and with caution. The exclusivity of cutaneous manifestations may be risky without the supporting history and diagnostic testing. This review contributes by systematically highlighting gaps in training, diagnostic resources, and interdisciplinary integration that have not been fully addressed in previous literature. Dermatology must be integrated into tropical medicine policy, practice, and research to ensure timely detection and management of infectious diseases in a fast-changing world. Furthermore, incorporating dermatological expertise into frontline healthcare can enhance early recognition of emerging infections and improve patient outcomes. Training clinicians to interpret subtle skin signs alongside other clinical data is essential for accurate and timely diagnosis. Future applications include developing targeted training programs, clinical decision support tools, and AI-assisted diagnostic systems that leverage dermatologic input. Ultimately, a collaborative approach that values dermatology strengthens the overall effectiveness of infectious disease surveillance and response.
